# The natural history of solitary post-nephrectomy kidney in a pediatric population

**DOI:** 10.1590/S1677-5538.IBJU.2018.0291

**Published:** 2019-12-17

**Authors:** Sánchez Basto Catalina, Puerto Niño Angie Katherine, Fernandez Nicolas, Castillo Mariangel, Espitaleta Vergara Zilac, Ana María Quintero Gómez, Pérez Niño Jaime

**Affiliations:** 1 Pontificia Universidad Javeriana, Bogotá, Colombia; 2 Departamento de Urología, Pontificia Universidad Javeriana, Hospital Universitario San Ignacio, Bogotá, Colombia; 3 Departamento de Nefrología, Hospital Universitario San Ignacio, Bogotá, Colombia; 4 Departamento de Epidemiología Clínica, Hospital Universitario San Ignacio, Bogotá, Colombia; 5 Universidad Simon Bolivar, Bogotá, Colombia; 6 Departamento de Nefrología Universidad del Bosque, Bogotá, Colombia; 7 Departamento de Urología, Fundación Santa Fe de Bogotá

**Keywords:** Solitary Kidney, Renal Insufficiency, Nephrectomy

## Abstract

**Introduction:**

Children with a solitary post-nephrectomy kidney (SNK) are at potential risk of developing kidney disease later in life. In response to the global decline in the number of nephrons, adaptive mechanisms lead to renal injury. The aim of this study was to determine the prevalence and time of onset of high blood pressure (HBP), proteinuria, glomerular filtration rate (GFR) disruption and renal tubular acidosis (RTA) in children with SNK.

**Materials and methods:**

After obtaining the approval from our institution's ethics committee, we reviewed the medical records of patients under 18 years of age who underwent unilateral nephrectomy between January 2005 and December 2015 in three university hospitals.

**Results:**

We identified 43 patients, 35 (81.4%) cases of unilateral nephrectomy (UNP) were due to a non-oncologic pathology and Wilm's tumor was identified in 8 (18.6%) cases. In patients with non-oncologic disease, 9.3% developed de novo hypertension, with an average time of onset of 7.1 years, 25% developed proteinuria de novo, with an average time of onset of 2.2 years. For GFR, 21.8% presented deterioration of the GFR in an average time of 3.4 years. Ten (43.5%) patients developed some type of de novo renal injury after UNP. Patients with oncologic disease developed the conditions slowly and none of them developed proteinuria.

**Conclusions:**

Taking into account the high rate of long term postoperative renal injury, it can be considered that nephrectomy does not prevent this disease. The follow-up of children with SNK requires a multidisciplinary approach and long-term surveillance to detect renal injury.

## INTRODUCTION

When compared to the general population, patients with a solitary kidney have an increased risk of developing chronic kidney disease (CKD) throughout life (1). There are reports of long-term outcomes but the results are variable and do not allow to confirm causality of kidney disease in the future (1–3).

Some case series report rates between 30 and 50% of kidney disease in these patients (4–8). However, the etiological burden that leads to end-stage kidney disease is unclear and there are no specific prognostic factors that can accurately predict this.

In response to the decreased numbers of nephrons, several adaptive mechanisms occur in the remaining ones which can manifest clinically as arterial hypertension (AHT), decreased glomerular filtration rate (GFR) and proteinuria. There are intraglomerular hemodynamic changes due to the initial hyperfiltration. It starts with intrarenal vasodilatation and glomerular hypertension which causes higher glomerular volume and surface. This inflicts a mechanical pressure on the hypertrophied podocytes, producing patches in the glomerular basement membrane which leads to a scarred Bowman's capsule and segmental sclerosis. Histopathological findings suggest focal and segmental sclerosis in these kidneys is what leads to long-term kidney disease (5, 6).

Currently, there are insufficient global and local statistics about the short, medium and long-term outcomes of the solitary kidney in the pediatric population.

The aim of this study was to determine the prevalence and time of presentation of hyperfiltration nephropathy, hypertension, proteinuria, decreased GFR and renal tubular acidosis (RTA) in pediatric patients with a solitary post-nephrectomy kidney. Thus, our goal is to expand the knowledge and evaluate the prognosis of these patients.

## MATERIALS AND METHODS

After obtaining the approval from our institution's ethics committee, we performed a retrospective analysis of data from pediatric patients who underwent unilateral nephrectomy between January 2005 and December 2015 at Hospital Universitario San Ignacio, Hospital Militar Central y Fundación Santa Fe de Bogotá in Bogotá, Colombia. Preoperative and postoperative conditions were analyzed: age, sex, weight, height, blood pressure, 24-hour urine total protein and/or urine protein to creatinine ratio, GFR by Schwartz formula, RTA, exposure to nephrotoxic drugs and the reason for performing the nephrectomy. BP and proteinuria values were adjusted according to age and GFR values were adjusted according to age and height.

With regard to BP, the Fourth Report on the Diagnosis, evaluation, and treatment of high bood pressure (HBP) in children was used: normal systolic blood pressure (SBP) <90th percentile, prehypertension SBP >90th percentile but <95th percentile or if BP exceeds 120/80mmHg even if below 90th percentile up to <95th percentile, HBP stage 1 (HPB 1) 95th percentile to the 99th percentile plus 5mmHg and HBP stage 2 (HPB 2) >99th percentile plus 5mmHg (9).

For proteinuria, we adopted the following classification: mild proteinuria 4-10mg/m2/h or urine protein to creatinine ratio (Pr/Cr) <1, moderate 10-40mg/m2/h or Pr/Cr 1-2 and massive: >40mg/m2/h or Pr/Cr >2 (10).

For GFR, we used the 2002 National Kidney Foundation Kidney Disease Outcomes Quality Initiative (KDOQI) classification of chronic kidney disease (CKD) from stages 1 to 5 (S I to S IV) (11).

And finally, for glomerular hyperfiltration we adjusted GRF according to age in preterm and full-term patients (10).

All of these variables were evaluated at the time of diagnosis, prior to unilateral nephrectomy, at the time of the surgical procedure, at 3, 6, 9, and 12 months and annually until possible.

It was defined if patients had improved, worsened or had no changes after surgery: Improved: partial or total amelioration of the preoperative disruption after surgery, a decrease in the stage of the disease and no development of new disruptions; Worsened: an increase in the stage of the disease or development of new disturbances post-operatively; and No changes: the stage of the disease remained the same and no development of new disruptions was observed.

The data of patients with oncologic nephrectomy indication and history of chemotherapy were evaluated separately since that factor could alter the outcomes. Patients who had stage 5 CKD and were undergoing dialysis were excluded from the post-operatively analysis since the natural history of their disease is already known.

Patients without follow-up were excluded.

Statistical analysis was performed using the Microsoft Excel^®^ 2016 program.

## RESULTS

A total of 43 patients entered the study. The mean age at diagnosis was 5.53 years (ante- natal-17 years). The percentage of men was 44% and women 56%.

Regarding the causes of nephrectomy, it was found that 35 (81.4%) were non-cancer patients and 8 (18.6%) were cancer patients.

## NON-CANCER PATIENTS

The main congenital anomalies of the kidney and the urinary tract (CAKUT) was reflux nephropathy in 42.9%, followed by ureteropelvic junction obstruction and ureterovesical junction obstruction with 20% each, followed by multicystic dysplastic kidney and ectopic ureter with 5.7% each and finally nephrolithiasis and nephroma with 2.9% each.

### Analysis by patients

46% of the patients had at least one preoperative disturbance such as BP, proteinuria, GFR or RTA 50% of them had just one condition and 50% two conditions.

28% of the patients with at least one preoperative condition had contralateral CAKUT.

The most frequent condition was proteinuria which was present in 50% of patients, the second one HBP present in 31% of patients and decreased GFR in 31% of them, the third one RTA in 25% and the least frequent condition was increased GFR in 12.5%.

After unilateral nephrectomy, 72% of the patients presented with some of the following disturbances: BP, proteinuria, GFR or RTA 56.5% of them had a history of kidney disease and 43.5% developed it de novo 80% of this last group had a normal contralateral kidney.

In the group with the de novo conditions, the most frequent disturbance was proteinuria in 50% of the patients, the second one decreased GFR in 30% of them, followed by HBP in 20%, increased GFR in 10% and TRA in 10% of the patients.

**Figure 1:**
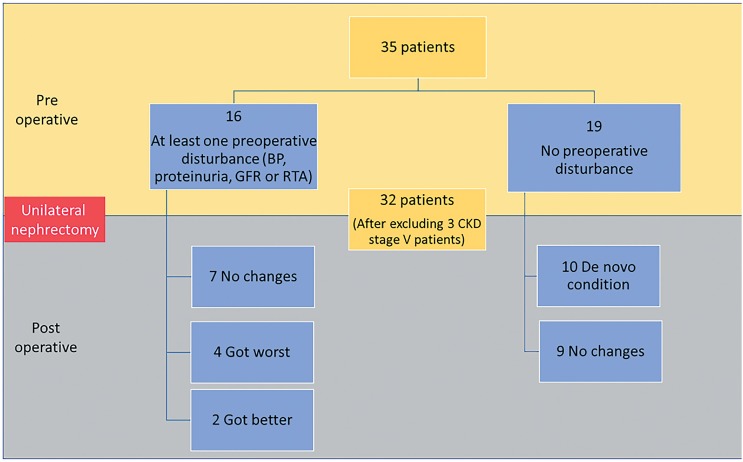
Overall of pre-surgical and post-surgical patient's condition.

Overall, 43.7% of the patients got worst after surgery, of which 71.4% developed some de novo condition such as BP, proteinuria, GFR or RTA and 29% had already a history of kidney disease (Figure-1).

### Pre-operative evaluation

Regarding BP, 83% of the patients had normal BP, 11% had HBP stage 2 and 3% had HPB stage 1. As for proteinuria, 17% had mild proteinuria and 6% moderate proteinuria 11% of the patients had RTA. Concerning GFR, 77% had CKD stage I, 8.5% CKD stage V, 8.5% increased GFR and 6% CKD stage II (Table-1).

**Table 1:** Pre and post- operative evaluation of patients who underwent unilateral nephrectomy for non-oncological condition.

Pre operative evaluation	Post operative evaluation					
Condition	Stage	N	%	Stage	N	%
HBP*	Normotensive	28	87.5	Normotensive	25	89
				Stage 1	3	11
				Stage 2	0	0
	Stage 1	1	3	Normotensive	0	0
				Stage 1	1	100
				Stage 2	0	0
	Stage 2	3	9.5	Normotensive	1	33.3
				Stage 1	1	33.3
				Stage 2	1	33.3
Proteinuria*	No proteinuria	24	75	No proteinuria	18	75
				Mild	2	8
				Moderate	3	13
				Massive	1	4
	Mild	6	18	No proteinuria	0	0
				Mild	4	66.6
				Moderate	2	33.3
				Massive	0	0
	Moderate	2	7	No proteinuria	2	100
				Mild	0	0
				Moderate	0	0
				Massive	0	0
	Massive	0	0	-		
RTA	No RTA	29	90.6	No RTA	27	84
				RTA	2	16
	RTA	3	9.4	No RTA	0	0
				RTA	3	100
GFR*∞	CKD S I	27	84.2	CKD S I	20	74.1
				CKD S II	4	14.8
				CKD S III	1	3.7
				CKD S IV	0	0
				CKD S V	0	0
				Increased GFR	2	7.4
	CKD S II	2	6.25	CKD S I	0	0
				CKD S II	2	100
				CKD S III	0	0
				CKD S IV	0	0
				CKD S V	0	0
				Increased GFR	0	0
	CKD S III	0	0			
	CKD S IV	0	0			
	Increased GFR	3	9.55	CKD S I	0	0
				CKD S II	0	0
				CKD S III	0	0
				CKD S IV	0	0
				CKD S V	0	0
				Increased GFR	3	100

* Age- adjusted

∞ Calculated by Schwartz.

### Post-operative evaluation

After excluding 3 patients with CKD stage V we evaluated the 32 remaining patients.

The mean follow-up time was 67.5 months (12-129.4 months), 78% of the patients were followed for more than 3 years.

Post-operative evolution compared to pre-operative state can be seen in Table-1.

Regarding BP, 9.3% of the patients got worst, all of them with normal pre-operative BP, with an average time of occurrence of 68.3 months (57, 4-109, 9 months). For patients with pre-operative HBP, 50% got better and 50% had no changes (Table-1).

In relation to proteinuria, 6% got better and 25% got worst, with an average time of occurrence of 27.3 months (8, 5–4, 13) 37.5% of the patients who got worst had contralateral CAKUT. Additionally, 25% of the patients developed de novo proteinuria (Table-1).

When RTA was evaluated, we found that 6.2% got worst and no one got better (Table-1).

As for GFR, 21.8% got worst with an average time of occurrence of 41.3 months (8, 06-94, 4 months). Only one patient got worst with an increase to S III CKD and higher, that patient had contralateral CAKUT. Only 2 patients increased their GFR and the ones that had pre-operative hyperfiltration remained the same (Table-1).

### Cancer patients

There were 8 oncological patients, two of them had no follow-up which is why they were excluded from the analysis.

### Analysis by patients

Fifty percent were female and 50% were male patients. As for laterality, 4 patients (66.6%) presented the tumor on the right side and 2 patients (33.3%) on the left side.

33.3% of the patients had at least one preoperative disturbance such as BP, proteinuria, GFR or RTA, 33.3% of them had HBP and proteinuria and 33.3% had no condition.

Overall, 4 patients (66.6%) got worst, three of them with a pre-operative condition and one of them developed a de novo condition which was HBP. Among the ones that got worst, one of them developed S II CKD, one of them developed RTA and one of them went from a stage 1 to a stage 2 HBP.

### Pre-operative evaluation

Two patients (33.3%) had HBP stage 1 and one patient (16.6%) HBP stage 2. As for proteinuria, one patient (16.6%) had mild proteinuria and one moderate proteinuria, none of them received pre- operative chemotherapy. There were no patients with RTA Regarding GFR, there were no patients with decreased GFR and just one with increased GFR.

### Post-operative evaluation

The mean follow-up time for these patients was 87.6 months.

Post-operative evolution compared to pre-operative state is shown in Table-2. Regarding HBP, two patients (33.3%) got worst, one presented with a stage 1 HBP and the other one with a stage 2 HBP. Two patients (33.3%) got better about this condition.

**Table 2:** Pre and post- operative evaluation of patients who underwent unilateral nephrectomy for oncological condition.

Pre operative evaluation				Post operative evaluation		
Condition	Stage	N	%	Stage	N	%
	Normotensive	3	50	Normotensive	2	66.6
				Stage 1	1	33.3
				Stage 2	0	0
HBP*	Stage 1	2	33.3	Normotensive	1	50
				Stage 1	0	0
				Stage 2	1	50
	Stage 2	1	16.6	Normotensive	0	0
				Stage 1	1	100
				Stage 2	0	0
Proteinuria*	No proteinuria	4	66.6	No proteinuria	4	100
				Mild	0	0
				Moderate	0	0
				Massive	0	0
	Mild	1	16.6	No proteinuria	1	100
				Mild	0	0
				Moderate	0	0
				Massive	0	0
	Moderate	1	16.6	No proteinuria	0	0
				Mild	1	100
				Moderate	0	0
				Massive	0	0
	Massive	0	0			
ATR	No RTA	6	100	No RTA	5	83.3
				RTA	1	16.6
	RTA	0	0			
GFR*∞	CKD S I	5	83.3	CKD S I	4	80
				CKD S II	1	20
				CKD S III	0	0
				CKD S IV	0	0
				CKD S V	0	0
				Increased GFR	0	0
	CKD S II	0	0			
	CKD S III	0	0			
	CKD S IV	0	0			
	Increased GFR	1	16.6	CKD S I	0	0
				CKD S II	0	0
				CKD S III	0	0
				CKD S IV	0	0
				CKD S V	0	0
				Increased GFR	1	100

* Age- adjusted

∞ Calculated by Schwartz.

In relation to proteinuria, there were no patients with de novo proteinuria, and the two patients with pre-operative proteinuria got better, one of them did not present this condition anymore and the other changed from moderate to mild proteinuria.

When RTA was evaluated, we found that one patient (16.6%) had developed RTA with an average time of 70.4 months.

As for GFR, one patient decreased his GFR to a stage II CKD, the rest of them had no change as to their pre-operative condition, the patient with increased GFR remained the same.

## DISCUSSION

### Non-cancer patients

The natural history of the single post-nephrectomy kidney has not been systematically specified in our population and there are not many studies about this in the literature.

In fact, in our population there is only one study about single kidney which evaluated unilateral multicystic renal dysplasia, nevertheless, it is a different condition from acquired single kidney so the results are not comparable (12).

In the world literature, the reference study about this is the KIMONO which is a retrospective study of renal injury markers performed in 206 children with congenital and acquired solitary functioning kidney (1).

Our study describes the evolution of an acquired single kidney pediatric population in Bogotá, Colombia, the conditions prior to unilateral nephrectomy and its outcome after the procedure.

We found that the most frequent cause of nephrectomy in pediatric population is reflux nephropathy followed by ureteropelvic junction obstruction, which is consistent with the world literature (1).

We found a significant number of patients with kidney disease during the follow-up phase after acquired solitary kidney, 43.7% worsened their initial condition and 43.5% developed a de novo condition. At the time patients underwent unilateral nephrectomy, almost half of them already had previous kidney disease which may indicate that this disease has a multifactorial component in relation to CAKUT.

However, post-operative changes play an important role in kidney disease because 71.4% of patients who worsened their condition had no previous kidney damage and 80% of those who had de novo disturbances had no CAKUT in the residual contralateral kidney. This suggests that long-term changes are part of the natural history of the acquired solitary kidney.

Given that in some proportion of patients undergoing unilateral nephrectomy, kidney function is compromised, and it can actually get worse, strict guidelines and parameters should be followed when deciding to perform this procedure.

The emergence of any disturbance such as BP, proteinuria, GFR or RTA during the post-nephrectomy phase in our study was 43.7% in an average time of 5.6 years of follow-up. It is a rate higher than the one reported on the literature where the average rate is 31-38, 1% in studies with mean follow-up of 10-14.9 years (1, 13, 14). It is possible that this difference could be due to the way the health system works in our country, where administratively it is difficult to ensure a strict and multidisciplinary follow-up to these patients.

### HBP

In our study, 14% of patients had some degree of hypertension. None of these patients worsened and 50% improved 9.3% developed de novo HBP. The literature describes HBP rates of 11% and 33.3% (1, 3, 5, 13–15). The high variability of the results could be explained by the lack of standardization of BP in each study. When BP is taken with ambulatory blood pressure monitoring compared to single office measurement, the diagnosis of hypertension can be increased by as much as 17% (15). The mean time of onset of hypertension in our study was 7.1 years, (86.3 months) similar to other studies that showed average times between 4.9 and 12 years (5, 13).

The large number of patients who developed hypertension and late onset hypertension portraits the importance of monitoring BP throughout childhood in single kidney patients.

### Proteinuria

Twinty tree percent of our patients had proteinuria prior to the surgical procedure, 25% developed it de novo, and 25% worsened during follow-up. In our series the occurrence of proteinuria is much higher than the one found in some investigations, which is between 6.7% and 19% (1, 13, 14). However, previous studies reported rates up to 70% (5). The big difference in the results may be secondary to cultural and demographic factors, since proteinuria is directly related to obesity, sedentary lifestyle, among others.

The average onset of de novo proteinuria was 2.2 years, which is faster when compared to another study that shows an average of 9.8 years (13). This difference is important, however, the larger studies and references such as the KIMONO cohort do not measure the onset time of this condition. More studies are necessary to define the real time (1).

The short time of proteinuria onset in our patients can also be explained by the fact that an important percentage (37.5%) of those who worsened, had CAKUT of the residual kidney.

### GFR

Twenty tree percent of patients had decreased GFR before nephrectomy and 21.8% got worst with an average time of 3.4 years. This value is higher than the one reported in the literature which is 6% with an average time of 6.4 years (13).

The KIMONO study reports that GFR deterioration is more evident after puberty but our study does not have a follow-up phase long enough to perform this type of analysis (1).

No patient developed end-stage kidney disease, which can be attributed to the follow-up time of our study, while KIMONO and others report that between 20-50% of single-kidney can develop this condition at their 30`s (1, 16).

### RTA

Given the low rate of appearance of RTA after nephrectomy, we consider that this is not a condition that is part of the natural history of the solitary post nephrectomy kidney patient.

### Cancer patients

As previously indicated, patients with oncological pathology deserve a different analysis given their history of chemotherapy. Nephrotoxicity due to chemotherapy is a controversial issue, some define that the decrease in kidney function secondary to chemotherapy is temporary and reversible, and is only present during chemotherapy (17); whereas other studies indicate that nephrotoxicity due to chemotherapy is multifactorial and does not depend solely on the chemotherapeutic agent used (18). The risk factors for these children may be inherent to their biological and medical conditions, such as decreased circulatory volume (diarrhea-vomiting), liver dysfunction, fluid sequestration and acute kidney disease. Likewise, there may be direct effects of cancer such as tumor lysis, hypercalcemia, disseminated intravascular coagulation, paraneoplastic glomerulopathies, among others (18).

A follow-up of 7.3 years (87.6 months) was carried out, similar to other case series reports published that indicate a follow-up time of 9.1 years (19). During this time, some patients developed progressive kidney disease, but in a smaller proportion compared to patients undergoing nephrectomy due to non-oncological pathology, this data agrees with the literature (19).

In addition, we noticed that patients with an oncological pathology developed a later decrease in GFR compared to patients with non-oncological pathology, 63.5 months vs. 41.3 months. Also, no patients with oncological pathology developed proteinuria compared to the 27.39% non-cancer patients who did.

This may suggest that those with oncological pathology have a healthy contralateral kidney with no underlying pathology to deteriorate it, a situation that is different from several cases of patients with non-oncological pathology.

### Study strengths and limitations

A characteristic that differ from the rest of the literature in regard to this topic, is that our study describes pre-operative and post-operative evolution of patients who underwent unilateral nephrectomy.

We believe that the results we show in this study are very meaningful, since they show the importance of long-term follow-up of single-kidney patients, especially in developing country health systems where it is not possible to ensure this, it is necessary to raise awareness of the importance of a strict and careful follow-up within the physicians and the health institutions that handle single-kidney patients.

The limitations of this study are that it is a retrospective study, with a significant but small number of patients compared to some other studies, and the follow-up does not exceed the adult age of the patients. Additionally, there is a selection bias, since it is a convenience sample of the patients taken to nephrectomy, which was reduced by including multiple health institutions.

Another limitation is that the development time of the conditions was defined at the time when it was documented in the follow-up meetings, but not when the patients developed it.

## CONCLUSIONS

Given the high rate of post-operative long-term kidney disease, it can be considered that nephrectomy does not completely prevent this disease which is the intention when the surgical procedure is performed; the surgical indications must be strict and maybe new surgical strategies should be considered.

Our case series reports the outcome of a significant number of single-kidney patients in our country, which contributes to statistics and allow us to give a prognosis to patients before performing a nephrectomy.

In the follow-up of children with a single kidney, a multidisciplinary approach is required, involving pediatricians, pediatric nephrologists and pediatric urologists, with strict long-term follow-up that includes the active search for conditions such as proteinuria, HBP and deterioration of TFG.
